# Improving Access to Care Through Out-of-Pocket Price Transparency Policy

**DOI:** 10.1093/geroni/igaf122.616

**Published:** 2025-12-31

**Authors:** Caroline Sloan

**Affiliations:** Duke University School of Medicine, Durham, North Carolina, United States

## Abstract

Technology Policy (ASTP) in the Department of Health and Human Services (DHHS) provides an opportunity to advance federal out-of-pocket price transparency regulation. As a primary care physician, I have long been frustrated by the inability to predict the costs of the medications I send to the pharmacy, limiting my ability to choose lower-cost and equally effective treatment options for my patients. In 2019, Medicare issued a regulation requiring Medicare Part D plans to provide “timely and accurate” out-of-pocket cost estimates to prescribers, via real-time benefit tools (RTBT). RTBTs are applications embedded in the electronic health records (EHR) that automatically display out-of-pocket cost estimates for prescription medications, prior authorization requirements, and any available lower-cost alternatives. Early research has found that patients whose clinicians use RTBTs are more likely to fill their prescriptions at the pharmacy and to save money on those prescriptions. However, clinicians at a third of US healthcare organizations do not have access to RTBTs. I am working on a team at ASTP that will update the RTBT regulation to account for some of the limitations in RTBT access and functionality encountered so far. Learnings will include: 1) understanding the rule-making process; 2) learning how to synthesize and respond to public comments from a variety of stakeholders; and 3) leveraging my clinical experience as an RTBT user and my research experience evaluating the impact of RTBTs on access to medications and clinical outcomes among adults with multimorbidity.

